# The self versus proxy report conundrum in juvenile idiopathic arthritis: implications for a cost-effectiveness analysis of integrated podiatry care

**DOI:** 10.1186/1757-1146-4-S1-P26

**Published:** 2011-05-20

**Authors:** Gordon J Hendry, Janet Gardner-Medwin, Debbie E Turner, Jim Woodburn, Paula K Lorgelly

**Affiliations:** 1 School of Biomedical and Health Sciences, University of Western Sydney, Sydney, Locked Bag 1797, Australia; 2 Glasgow Caledonian University, Glasgow, G4 0BA, UK; 3 University of Glasgow, Glasgow, G12 8QQ, UK; 4 Monash University, Victoria, 3800, Australia

## Background

JIA is associated with impaired health-related quality of life (HRQoL). Proxy reporting of health outcomes such as HRQoL is often required in the paediatric setting. However there is uncertainty regarding whether self- or proxy- reported HRQoL should be used in health economic analyses, as perceptions of well-being may differ between parent and child. The aim of this study was to estimate levels of agreement and association between self- and proxy-reported HRQoL in JIA.

## Methods

The EQ5D is a generic measure of HRQoL commonly used in economic evaluations. It has 2 components; 1) EQ5D profile comprises of 5 domains; mobility, self-care, usual activities, pain, and anxiety (scored as severe, moderate, or no problems); and 2) a 100mm visual-analogue-scale (VAS). EQ5D profiles were used to calculate a weighted utility index (worst health=-0.59, best health=1) based on a tariff derived from a UK population sample. The EQ5D was self- and proxy-completed independently by JIA patient-parent/guardian pairs in the paediatric rheumatology multi-disciplinary foot clinic (n=40). Agreement for the EQ5D profile items was estimated using Cohen’s linear-weighted kappa (κ_w_) (>0.4=moderate agreement) and 95% confidence intervals. Agreement for the utility index and VAS was estimated using the intraclass correlation coefficient (ICC) (≥0.4-0.75=fair to good agreement) with 95% confidence intervals. Consistency was estimated using Kendall’s Tau (τ) and Pearson’s correlation coefficient (r) (≥0.4-0.6=moderate-strong consistency).

## Results

Self- versus proxy- agreement was moderate for self-care (κ_w_=0.44) and usual activities (κ_w_=0.44), but less than moderate for mobility (κ_w_=0.29), pain (κ_w_=0.29), and anxiety (κ_w_=0.20). There was good agreement for EQ5D VAS (ICC=0.59), but poor agreement for the utility index (ICC=0.23). The consistency of HRQoL reporting was moderate for self-care (τ=0.47) and usual activities (τ=0.44), but weak for mobility (τ=0.31), pain (τ=0.33), and anxiety (τ=0.23). Consistency was good for EQ5D VAS (r =0.59) but weak for utility index (τ=0.28).

## Conclusions

The levels of agreement and association between patients with JIA and parent-proxies are at best moderate for all EQ5D measures. This has important implications for cost-utility analyses using QALYs derived from the EQ5D utility index. Further research is required to determine feasibility of using self- and proxy reported outcomes in cost-effectiveness analyses of podiatric interventions.

